# Alkaloids from* Caliphruria subedentata* (Amaryllidaceae) as Regulators of AChE, BuChE, NMDA and GSK3 Activity: An In Vitro and In Silico Approach for Mimicking Alzheimer´s Disease

**DOI:** 10.1007/s11064-025-04354-6

**Published:** 2025-03-08

**Authors:** Willian Orlando Castillo Ordoñez, Nilza Velasco Palomino, Patricia Eugenia Vélez Varela, Ivon Bolaños Martínez, Levy Bueno Alves, Silvana Giuliatti

**Affiliations:** 1 Departamento de Biología, Facultad de Ciencias Naturales-Exactas y de la Educación, Universidad del Cauca, Cra 2 No 2N-57, 19003 Popayán-Cauca, Colombia; 2 Departamento de Estudios Psicológicos, Universidad Icesi, Cali, Colombia; 3 Department of Genetics, Ribeirão Preto Medical School, University of São Paulo – USP, São Paulo, Brazil

**Keywords:** Cholinergic activity, Molecular docking, Molecular dynamic, Alkaloids, ADMET properties

## Abstract

Patients with Alzheimer’s disease (AD) have two types of abnormal protein buildups: amyloid plaques and neurofibrillary tangles, in addition to the early synaptic dysfunction associated with the enzymes acetylcholinesterase (AChE) and butyrylcholinesterase (BuChE). Impairment of the glutamatergic system is also crucial for neuronal survival, as it can cause synaptic dysfunction that overstimulates glutamate receptors, especially N-methyl-d-aspartate receptors (NMDARs). Another protein affecting neuronal health is glycogen synthase kinase-3 (GSK3), a widely preserved serine/threonine protein kinase linked to neuronal disorders, including AD. In recent years, alkaloids from the Amaryllidaceae have received great attention for their known anticholinergic activity, as well as their antioxidant, antigenotoxic, and neuroprotective properties. In this context, the identification of compounds capable of interacting with different targets involved in AD provides a possible new therapeutic strategy. In this study, we conducted a combination of in vitro and in silico approaches to identify the potential of *C. subedentata* in regulating key proteins involved in AD. Viability and neuroprotection assays were performed to evaluate the neuroprotection exerted by *C. subedentata* extract against neurotoxicity induced by Aβ (1–42) peptide and Okadaic acid in SH-SY5Y cells. Computational methods such as docking and molecular dynamic and viability therapeutic analysis were conducted to explore the interaction of alkaloids from *C. subedentata* with target proteins (AChE, BuChE, NMDA, and GSK-3) involved in AD. Our findings show that C. *subedentata* extract exerts neuroprotective effects against neurotoxic stimuli induced by Aβ (1–42) peptide and Okadaic acid. In addition, in silico approaches provide insight into how *C. subedentata* extract alkaloids interact with key proteins involved in AD. These findings provide insights into the potential therapeutic effects and action mechanisms of these alkaloids. We hope these rapid findings can contribute as a bridge to the identification of new molecules with the potential to counteract the effects of AD.

## Introduction

Alzheimer’s disease (AD) is a devastating neurodegenerative disease, and the most common form of dementia among the elderly population, worldwide affects nearly 50 million people [1]. AD is an illness manifested by a number of pathological and clinical features, which includes amyloid-beta (Aβ) peptide deposits, hyperphosphorylation microtubule-associated tau protein by glycogen synthase-3(GSK3) activity, which leads to the formation of neurofibrillary tangles (NFTS) [2], dystrophic neurites, cholinergic impairment and neuronal death. Clinically, patients with AD exhibit a gradual loss of memory, and the ability to carry out daily activities. Additionally, as the disease progresses, patients will have trouble, such as language impairment, thinking skills, depression, hostile behavior, hallucination and psychosis, and will finally need complete care from caregivers during the end stage of AD [3–6].

Although AD has been studied for over a century, there is not treatment that gets to change the course of the disease. To date, cholinergic inhibitors such as donepezil, rivastigmine and, galantamine have been drugs approved by the US Food and Drugs Administration (FDA) for its treatment; however, these drugs provide symptomatic treatment but do not alter the course of disease. Likewise, commercially available drugs used for its symptomatic treatment show side effects such as gastrointestinal disturbance, vomiting, diarrhea, muscle aches, heartburn, headache, hepatotoxicity, and shorter half-life. The dysfunction of the cholinergic system in memory processing and storage is the base of the commonly accepted cholinergic hypothesis [7]. According to the hypothesis, in AD there is a loss of cholinergic neurons and nicotinic acetylcholine receptors (nAChRs), which correlated with cognitive decline observed in the AD patients [8]. Acetylcholinesterase inhibitors (AChEIs) enhance cholinergic neurotransmission by inhibiting the AChE enzyme, which is responsible for breakdown of the neurotransmitter acetylcholine (ACh), and this way, prolongs its action at the synaptic cleft. However, recently it has also been shown that another type of cholinesterase known as butyrylcholinesterase (BuChE) acts as a co-regulator of cholinergic neurotransmission by hydrolyzing ACh with a mechanism of degradation of the neurotransmitter similar to AChE. All of this is due to the functional and structural similarity of these enzymes [9–11]. On the other hand, dysfunction in the glutamatergic system seems to be critical for the survival of neurons. Glutamate is the most abundant excitatory neurotransmitter in the mammalian Central Nervous System (CNS). The great majority of the excitatory neurotransmission is mediated by glutamate and its receptors, as N-methyl-d-aspartate (NMDA) [12]. The activation of synaptic NMDA can both promote neuronal health and kills neurons by activating Ca^2+^ dependent transcription factors such as cyclic-AMP response element-binding protein (CREB) and suppresses caspases and apoptotic pathway. So anyhow, excessive stimulation of glutamatergic signaling leads to excitotoxicity, which is mediated by excessive Ca^2+^ entry through NMDA receptor, resulting in neuronal damage or death [13, 14]. Pharmacologically, memantine is an uncompetitive NMDA antagonist approved for the treatment of moderate to severe AD. Its mechanism of action differs from the major alternative therapies in AD, which are all AChEIs. Memantine blocks NMDA receptor by trapping it in the open conformation. The impairment of cholinergic neurons probably occurs early in the disease whilst glutamatergic damage and excitotoxic deregulation occurs late in the course of disease [15]. Nowadays, memantine and donepezil combination in moderate to severe stages of AD is ongoing.

The multifactorial condition of AD creates considerable difficulty in identifying an effective treatment; however, AChE inhibitory activity is among the most relevant therapies for decreasing the effects associated with the pathology. Nature provides a rich source of potential AChE inhibitors. In recent years, there has been a growing interest in the search of multifunctional compounds, which may represent an important pharmacological advance in the fight against the disease. In the light of this, the combination of phytochemical compounds by synergistic effect could interfere simultaneously at different levels of the neurotoxic pathways. Plants contain compounds with antigenotoxic, antioxidant, and anti-inflammatory activities, as well as the ability to inhibit AChE [16, 17].

Among many natural products, Amaryllidaceae plants and their alkaloids have attracted considerable attention due to their wide biological activity; including antiviral, anti-cancer and antigenotoxic, in addition to AChE and BuChE inhibitory activity [18–20]. Nowadays, FDA approves galantamine obtained from various Amaryllidaceae plants for clinical therapy. Galantamine has a dual mechanism of action, since it allosterically modulates nicotinic ACh receptors (nAChRs) and inhibits AChE. Additionally, galantamina modulates non-amyloidogenic processing of the amyloid precursor protein (APP) by inhibiting BACE (beta-site APP cleaving enzyme) [21–23]. This has motivated the screening of new metabolites from the Amaryllidaceae, as natural products represent a significant source of diverse molecules with biological potential. These metabolites can serve as guide compounds for the design and synthesis of new drugs. *Caliphruria subedenta* (commonly known Varita de San José) an endemic species of Colombia belonging to the Amaryllidaceae family is currently classified as endangered. Previous analysis by gas chromatography coupled with mass spectrometry (CG-MS) made it possible to isolate and identify alkaloids such as galantamine (24.77%) followed by maritidine (21.45%), tazettine (12.45%), galantindol (8.45%), haemanthamine (5.45%), ismine (4.27%), narwedine (3.79), lycorine (2.73), deoxytazettine (2.32%), clidantine (1.18%), 5,6-dihydrobicolorine (0.78%), 6-methoxypretazzetine (0.72%), epimacronine (0.647%) and kirkine (0.40%) [24]. The *C. subedentata* extract has been shown to exhibit neuroprotective effects by decreasing necrotic cell death, DNA damages, and mitochondrial morphological alterations induced by Aβ (1–42); besides, the extract exerted AChE inhibition activity [25]. However, it is not known which metabolites present in the extract are responsible for neuroprotective effects.

Among the newer approaches in this research field in silico analysis is successfully used to identify, design, and predict novel therapeutic targets. Both molecular docking and molecular dynamic are widely used in the discovery and optimization of novel compounds with affinity for a target. These methods also facilitate the correlation of biological activity with chemical constituents, which can provide valuable insights for predicting biological activity [26, 27]. In this context, computational methods based on protein–ligand interaction were adopted to explore the inhibitory activity exerted by some alkaloids belonging to the Amaryllidaceae family on four major protein targets (AChE, BuChE and NMDA and GSK3) by applying in vitro and in silico analysis complemented with therapeutic viability approaches.

## Methodology

### Plant Collection and Extract Preparation

Dormant bulbs of *Caliphruria subedentata* were recollected from the Botanical Garden “Alvaro José Negret”, affiliated with the Universidad del Cauca, in Popayán-Colombia. The plant material was examined and identified by Professor Bernardo Ramiro Ramírez, and a voucher specimen (N° 0,027,587) was deposited in the Herbarium of the University of Cauca, Colombia. The ethanolic extract of *C. subedentata* was prepared using ultrasound-assisted extraction. Powdered plant material was placed in a conical flask containing 0.25 L of 70% ethanol and sonicated for four hours in a Branson 2510 ultrasonic bath. After cooling to approximately 25 °C, the mixture was filtered, and the filtrate was dried under vacuum at 40 °C using a rotary evaporator. The dried extract was dissolved in water, filtered through a 0.2 μm membrane filter for use in subsequent experiments [25]. The chemical composition of the extract was characterized using gas chromatography-mass spectrometry [24].

### Materials and Methods

#### Materials

The Aβ (1–42) and Okadic Acid (OA) were purchased from Sigma-Aldrich, St. Louis, MO. The Aβ (1–42) peptide was prepared as previously described [25]. OA was dissolved in dimethyl sulfoxide (DMSO) at 100 µM as a stock solution.

### Culture of SH-SY5Y Cells

SH-SY5Y cells are a well-established human neuroblastoma cell line widely used in neurobiology research. They are particularly valuable for studying various neurological conditions, including Alzheimer’s disease, Parkinson’s disease, cerebral ischemia, neurotoxicity, and epilepsy. Due to their neuronal characteristics, SH-SY5Y cells serve as reliable models for investigating aspects of neuronal function and dysfunction [28, 29]. For the experiments, SH-SY5Y cells were cultured in a 1:1 mixture of DMEM/HAM-F-10 medium (Sigma-Aldrich, St. Louis, MO, USA), supplemented with 10% Fetal bovine serum, penicillin (100 U/mL) and streptomycin (100 µg/mL) (Sigma-Aldrich, St. Louis, MO, USA). The cell cultures were maintained at 37 °C in a humid atmosphere containing 5% CO2 and 95% air.

### Viability and Neuroprotection Assays

To assess cell viability and the neuroprotective effects of *C. subedentata* extract against Aβ (1–42) and OA-induced neurotoxicity in SH-SY5Ycells, the neutral red uptake assay was employed. This assay is based on the accumulation of neutral red, a weak cationic dye in the lysosomal organelles of viable cells [30]. In one set of experiments, cells (9 × 10^4^) were treated with *C. subedenta* extract at concentrations of 6.25, 12.5 and 25 µg/mL for 24 h. Following this, the medium was replaced with fresh culture medium, and the cells were exposed to the Aβ (1–42) (10 µM) or OA (30 nM) for an additional 24 h. In another set cells (9 × 10^4^) were co-treated with Aβ (1–42) (10 µM) or OA (30 nM) and the *C. subedentata* extract (6.25, 12.5 and 25 µg/mL) simultaneously for 48 h. After the treatments, the medium was removed, and 100 µl of neutral red solution was added to each well and incubated at 37 °C for 3 h. The supernatant was then discarded, and the dye retained by viable cells was extracted with 100 μl of an acetic acid: ethanol solution for 20 min. Absorbance was measured at 540 nm using a microplate reader.

### Docking Molecular and Therapeutic Viability

#### Protein Preparation

The X-ray crystallographic structures of the target proteins rhAChE (4EY6), BuChE (5DYW), NMDA (1PBQ) and GSK3 (4ACG) were downloaded from Protein Data Bank (PDB) [31]. Hydrogen atoms were added to the protein, and unnecessary water molecules and co-crystallized ligands were removed from the binding site. Protein preparation was carried out using the GOLD ® (V 5.1) protocol [32]. The binding sites, which included the co-crystallized ligands were used to define the active site of each target.

#### Selection of Ligands

The molecules of interes along with their identification codes were obtained from public repository PuBuChEm as follow: ismine (CID: 188,957), kirkine (CID: 399,198), maritidine (CID: 11,185,307), tazettine (CID: 271,607), anhydrolycorine (CID: 619,567), homolycorine (CID: 160,473), narwedine (CID: 10,356,588), galanthindole (CID: 71,369,229), 6LQ (CID: 56,962,316), ATP (CID: 5957), tacrine (CID: 1935), memantine (CID: 4054) and galantamine (CID: 9651) [33]. All 3D structures used for the docking studies were downloaded in SDF file format. Ligand preparation was also performed using GOLD ® (V 5.1). These alkaloids have previously been identified in *C. subedentate* (Amaryllidaceae) [24]. Additionally, the neuroprotective activity of the total *C. subedentata* extract has been reported in vitro [25].

In this study, docking simulations were conducted using the GOLD software (version 5.1). For each ligand, multiple poses were generated, and the CHEMPL scoring function was used to rank these poses based on their predicted binding affinity. The pose with the highest CHEMPL score was selected for further analysis, as it represents the most favorable binding conformation according to the scoring algorithm. To ensure consistency and reliability, the selected poses were visually inspected to confirm key interactions with the residues of the binding sites. Prediction of the pharmacokinetic properties of alkaloid ligands was performed by SwissADME platform [34] and ProTox-II [35]. Additionally, the PLIP [36] was used to analyze the interface of the ligands in the targets binding site. Visual inspection of the protein/ligand complexes was done by using PyMol software (Version 2.0).

### Molecular Dynamic

The GROMACS package version 2019.3 [37] was used for the molecular dynamic simulations of the complexes. The CHARMM36 force field was applied for the protein and solute [38]. Hydrogens were added to each ligand, considering the protonation state at pH 7.4, using the Avogadro software [39]. Ligand parameters were then derived using the CGennFF server [40]. Ensuring compatibility with the CHARMM36 force field. The complexes were centered in a box with a 10 Å distance from the edge and solvated in a cubic box containing TIP3P water molecules. The system was neutralized by adding the appropriate number of sodium (Na^+^) and chloride (Cl^−^) ions to achieve an ionic concentration of 0.15 M. Energy minimization was carried out using the steepest descent method with a maximum force of 1000 kJ/mol^−1^. nm for 50,000 steps. After minimization, the system was equilibrated in two steps: first in a canonical NVT (number of particles, volume and temperature) ensemble followed by an isothermal-isobaric NPT (number of particles, pressure and temperature) ensemble. NVT equilibration was carried out at a constant temperature of 300 K for 500 ps while NPT equilibration was performed at a constant pressure of 1 bar and temperature of 300 K for 500 ps. The production phase was carried out at 300 K for 100 ns, a duration commonly used in molecular dynamics studies to ensure sufficient sampling of the dynamic behavior of the complexes. An integration time step of 2 fs was applied for all simulations, with trajectories saved every 0.5 ns for subsequent analysis.

#### Binding Free Energy Calculation

The obtained MD trajectories were used to estimate the binding free energy and residue energy decompositions using molecular mechanics Poisson–Boltzmann surface area (MM/PBSA) protocols implemented in the *g_mmpbsa* package [41]. All frames from the last 10 ns of the production run for each complex were extracted and used for the MM/PBSA calculations. The standard output included the binding energy terms for the three simulations, as well as the binding free energy, calculated using the following equations [41].

1 $$\Delta {\text{G}}_{{{\text{binding}}}} = {\text{ G}}_{{{\text{complex}}}} - \, \left( {{\text{G}}_{{{\text{protein}}}} - {\text{ Gl}}_{{{\text{ligand}}}} } \right)$$

The G_complex_ is the total free energy of the protein − ligand complex and G_protein_ and G_ligand_ are total free energies for protein and ligand respectively.

2 $${\text{Gx}} = \, < {\text{ E}}_{{{\text{MM}}}} > \, - {\text{ TS }} + \, < {\text{G}}_{{{\text{solvatation}}}} >$$

where x is the protein. ⟨ E_MM_⟩ is the average molecular mechanics' potential energy. TS refers to the entropic collaboration of the free energy where T (temperature) and S (entropy) and G_solvation_ is the free energy of solvation.

3 $${\text{E}}_{{{\text{MM}}}} = {\text{ E}}_{{{\text{bonded}}}} + {\text{ E}}_{{{\text{nonbonded}}}} = {\text{ E}}_{{{\text{bonded}}}} + \, \left( {{\text{E}}_{{{\text{vdW}}}} + {\text{ E}}_{{{\text{elec}}}} } \right)$$

The E_bonded_ is bonded interactions consisting of bond, angle, dihedral and improper interactions. The nonbonded interactions (E_nonbonded_) include both electrostatic (E_elec_) and van der Waals (E_vdW_).

4 $${\text{G}}_{{{\text{solvatation}}}} = {\text{ G}}_{{{\text{polar}}}} + {\text{ G}}_{{{\text{nonpolar}}}}$$

The solvation free energy is expressed G_polar_ and G_nonpolar_ that are the electrostatic and nonelectrostatic contributions.

## Statistical Analysis

The results are expressed as means ± standard deviations (S.D) obtained from three independent experiments for each assay, each performed in triplicates. Statistically significant differences were assessed using a one-way analysis of variance (ANOVA) followed by Bonferroni´s post hoc test, utilizing GraphPad Prism Version 5.01 for Windows (GraphPad Software Inc. USA). A p-value of < 0.05 was considered statistically significant.

## Results and Discussion

### Chemical Composition of the Extract of *Caliphruria subedentata*

The chemical composition of *C. subedentata* extract was previously characterized in studies by Cabezas et al. and Castillo et al. [24, 25]. The principal compounds identified and isolated from the extract include galantamine (24.77%), maritidine (21.45%), tazettine (12.45%), galantindol (8.45%), haemanthamine (5.45%), ismine (4.27%), narwedine (3.79%), lycorine (2.73%), deoxytazettine (2.32%), and kirkine (0.40%). Additional minor metabolites identified were trisphaeridine, 11,12-dehydroanhydrolycorine, homolycorine, and anhydrolycorine. The concentrations of *C. subedentata* extract used in the test were consistent with those reported in early studies [25].

### *Caliphruria subedenta* Extract Offers Protection Against Neurotoxicity Induced by Aβ (1–42) Peptide and Okadaic Acid

**Fig. 1:**
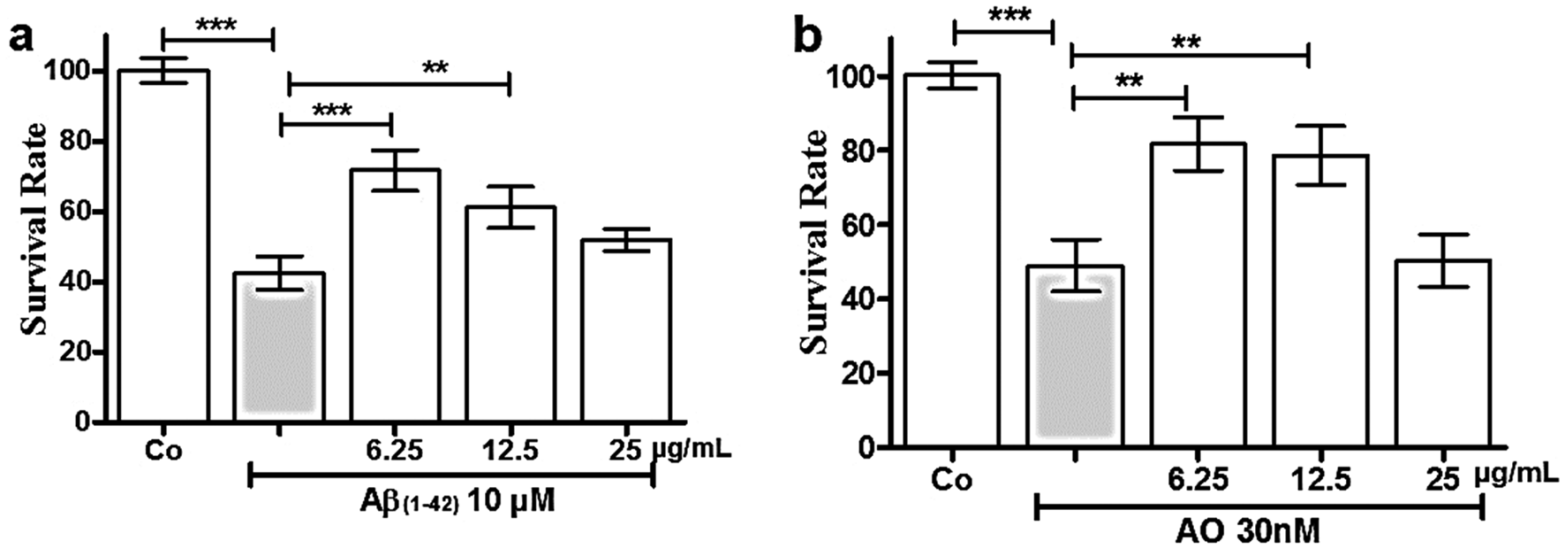
*C. subedentata* extract offers protection against Aβ(1–42) and OA-induced neurotoxicity in the human neuroblastoma cell line SH-SY5Y. SH-SY5Y cells were treated for 24 h with increasing concentration (6.25, 12.5 and 25 µg/mL) of *C. subedentata* extract, then, the cells were incubated with **a** Aβ(1–42) (10 µM) and **b** OA (30 nM) to induce neurotoxicity for 24 h. Data are means ± standard deviation (S.D) calculated for three independent experiments each one performed in triplicate. ^**^, *p* < *0.01* and ^***^, *p* < *0.001* in comparison with injured cells by Aβ (1–42) and OA. Co, cells not treated

Aβ (1–42) peptide and OA are well-established neurotoxic agents used in various in vitro and in vivo models. Neurotoxicity induced by these compounds is widely accepted as a model for mimicking neuronal death associated with AD. Aβ (1–42) peptide, a biochemical hallmark of neurodegeneration, is believed to result from aberrant processing of amyloid precursor protein (APP). In contrast, OA, a toxin produced by marine algae, inhibits protein phosphatases, leading to increased tau phosphorylation and neuronal death. To evaluate whether pretreatments or co-treatments with *C. subedenta* extract provides significant neuroprotection against these neurotoxic stimuli, SH-SY5Y cells were treated for 24 h with *C. subedenta* extract at concentrations of 6.25, 12.5, and 25 µg/mL, followed by 24 h exposure to Aβ (1–42) (10 µM) or OA (30 nM). Under our experimental conditions, pre-treatments with *C subedentata* extract at 6.25 and 12.5 µg/mL significantly increased the cell survival rate in response to Aβ (1–42) and OA-induced neurotoxic (Fig. 1).

**Fig. 2:**
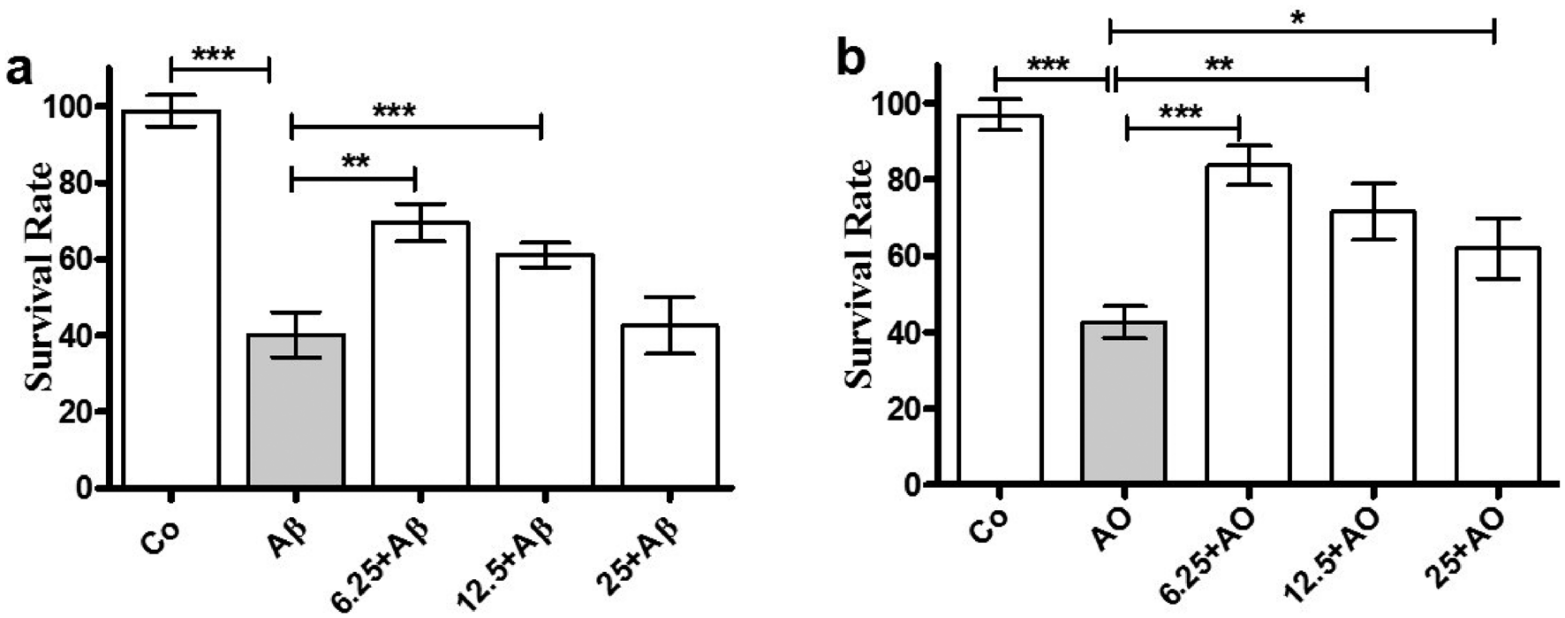
Simultaneous treatments with *C. subedentata* extract and neurotoxic stimuli decrease the neurotoxicity in SH-SY5Y cells. SH-SY5Y cells were incubated during 48 h with increasing concentration (6.25, 12.5 and 25 µg/mL) of *C. subedentata* plus Aβ (1–42) (10 µM) (**a**) or OA (30 nM) (**b**). Data are means ± standard deviation (S.D) of triplicate of three different experiments. ^*^, *p* < *0.05,* ^**^, *p* < *0.01* and ^***^, *p* < *0.001* in comparison with injured cells by Aβ (1–42) and OA. Co, cells not treated

Under different experimental conditions, SH-SY5Y cells were treated simultaneously with *C. subedenta* extract and neurotoxic stimulus (Aβ (1–42) at 10 µM or OA at30 nM. As shown in Fig. 2, both Aβ (1–42) and OA significantly decreased cell survival (*p* < *0.001*). However, co-treatment with the extract at concentrations of 6.25, 12.5 and 25 µg/mL led to significant increases in cell survival compared to the control.

These results demonstrate that both Aβ (1–42) and OA significantly reduce the proliferative capacity of SH-SY5Y cells. The pathological hallmarks of AD include the deposition of Aβ (1–42) and the accumulation of hyperphosphorylated tau protein [42]. In the context of Aβ (1–42)-induced cell death, Aβ is recognized as the primary constituent of senile plaques in AD and plays a pivotal role in its pathogenesis. Increasing evidence indicates that Aβ induces oxidative damage to proteins, disrupts calcium homeostasis by penetrating the mitochondrial matrix, and progressively accumulates to induce mitochondrial stress. This stress interferes with enzymatic activity and impacting neuronal bioenergetics [43, 44]. Consequently, these process lead to ATP depletion, which is critical for mitotic functions and cell death pathways. Additionally, Aβ exacerbates genotoxic damage in SH-SY5Y cells [25, 45, 46].

OA serves as a robust model for mimicking tau hyperphosphorylation in AD. This toxin inhibits phosphatases, displaying the highest affinity for PP2A followed by PP1 and PP2B. Its effects include inducing hyperphosphorylation in both in vivo and in vitro [47, 48]. Tau proteins isolated from AD brains exhibit over 40 phosphorylation sites with enzymes such as glycogen synthase kinase (GSK-3β) and protein phosphatase 2A (PP2A) playing critical roles in tau phosphorylation and dephosphorylation respectively [49]. In SH‐SY5Y cells, phosphorylation of GSK-3β at Ser9 reduces its activity, while phosphorylation at Tyr216 increases it [50]. The association between tau hyperphosphorylation and neuronal death through PP2A inhibition and GSK3 activation by OA has been documented in cortical and hippocampal neuron cultures, as well as SH-SY5Y cells [51–53]. Conversely, GSK3 inhibitors have been shown to reduce tau hyperphosphorylation. Research into the mechanisms of AChEIs has expanded beyond AChE inhibition to include their ability to mitigate cell damage by decreasing Aβ deposition [54]. Regarding the neuroprotective mechanisms of *C. subedentata* extract during pre and co-treatment against Aβ (1–42_)-_induced neurotoxicity, it has been reported that post-treatments with the extract reduces neuronal death by inhibiting AChE, decreasing genotoxic damage, and regulating the mitochondrial morphology [25]. Since neuronal homeostasis depends closely on mitochondria motility and function-crucial for differentiation processes [55, 56], it is plausible that *C. subedentata* alkaloids influence key energetic pathways involved in differentiation. Additionally, treatments with *C. subedentata* have been shown to promote neural differentiation effectively, likely through epigenetic regulation involving, with histone deacetylases [57]. Overall, our findings demonstrate that *C. subedentata* extract exerts protective effects during pre-treatment, co-treatment, and post-treatment [25] against neurotoxic stimul. However, these cannot be attributed solely to alkaloids, as non-alkaloid compounds may also contribute to their neuroprotective action. These results underscore the importance of exploring strategies aimed at not only treating degenerative pathologies but also on preventing them.

### Molecular Docking and Therapeutic Viability

Nowadays, due to the impact of AD on patients, caregivers and society, there is a growing need to find treatment or cure, and this way, reducing worldwide the effects of pathology. Because prevalence of AD is strongly associated with increasing age, it is expected that this dementing disorder will pose huge challenges to public health and elderly care systems worldwide [58]. To date, drug discovery efforts for AD have achieved limited success, primarily in the development of symptomatic treatments. These include AChEIs such as donepezil, rivastigmine, galantamine and memantine, an N-methyl-D-aspartate receptor (NMDA) antagonist. While, these drugs offer palliative benefits, they do not alter the progression of the disease. This failure in developing effective treatments can be attributed to the complex, multifactorial nature of AD pathogenesis and the absence of adequate and validated biomarkers, which significantly hinder the development of targeted therapies [3].

In this study, molecular docking was employed to evaluate the binding affinity between four targets (AChE, BuChE, NMDA and GSK3) and a selection of alkaloids ismine, kirkine, maritidine and tazettine, anhydrolycorine, homolycorine narwedine and galanthindole. Positive controls used for comparison included galantamine, tacrine, memantine, 6LQ and ATP. All tested alkaloids were capable of forming stable complexes. The docking scores for each complex are summarized in Table 1. Among the alkaloids anhydrolicorine, maritidine and galanthindole exhibited the highest average docking scores (60.08, 59.74, and 58.69 respectively), surpassing the score of galantamine (57.97). For the BuChE target, all alkaloids, except for ismine, demonstrated mean docking score values exceeding that of the positive control tacrine (52.50). In the case of the NMDA receptor, all ligands achieved higher mean scores values compared to memantine (40.67). Regarding the GSK3 target, kirkine exhibited the highest docking score among the alkaloids (57.56), however, this value did not surpass the scores observed for the positive controls 6LQ (83.77) and ATP (62.35).

**Table 1:** GOLD Mean scores. Molecular docking scores of ligands with target receptors

Ligands	CHEMPL mean score			
	AChE	BuChE	NMDA	GSK3β
	Galantamine	Tacrine	Memantine	6LQ, ATP
Positive control	57.97	52.50	40.67	83.77 62.35
Ismine	56.26	52.50	55.88	46.64
Kirkine	55.75	55.78	50.33	57.56
Maritidine	58.69	56.13	54.35	45.19
Tazettine	51.35	58.99	46.63	48.87
Anhydrolycorine	60.08	52.85	50.52	54.01
Homolycorine	55.13	59.61	57.87	44.60
Narwedine	52.79	55.05	55.36	43.80
Galanthindole	59.74	63.24	55.92	51.69

Gold scores are unitless values that represent the fitness of ligands. Higher scores indicate greater affinity of the ligands for the target protein

**Fig. 3:**
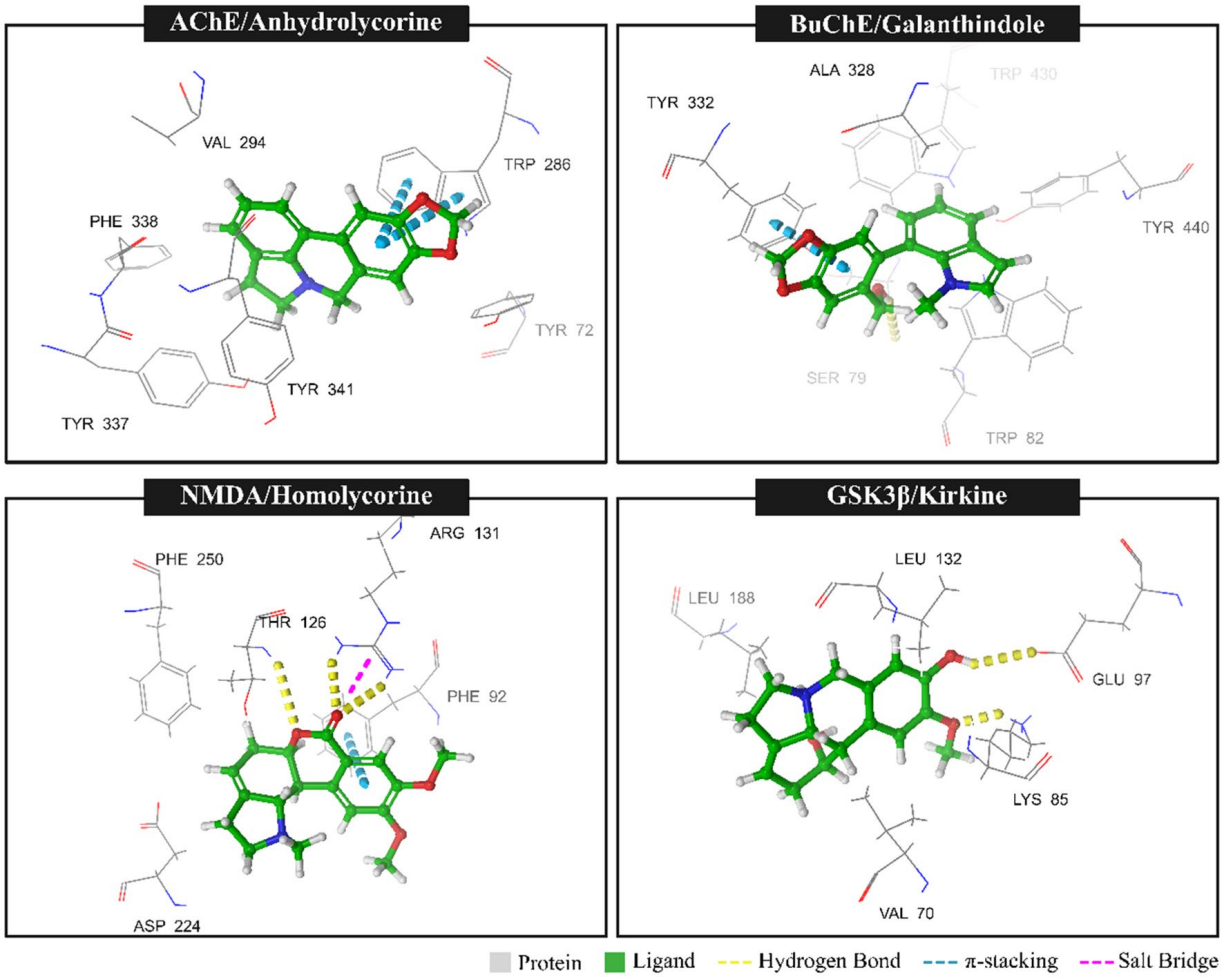
Top: Molecular interaction between ligands and targets

To evaluate the stability of the complexs, we focused on the ligands with the highest docking scores: anhydrolycorine/AChE = 60.0; galanthindole/BuChE (63.24); homolicorine/NMDA (57.87) and kirkine/ GSK3-β (57.56). The results of the protein–ligand interactions for the best pose of each ligand are summarized in Table 2 and illustrated in Fig. 3. The analysis revealed distinct interaction patterns across the complexes. The GS3K/Kirkine complex exhibited the highest number of hydrophobic interactions (six) and hydrogen bonds (three), highlighting its strong binding stability. In contrast, anhydrolycorine/AChE complex demonstrated the greatest number of pi-stacking interactions (three), emphasizing its unique model of binding. Notably, the NMDA/homolycorine complex showed a balanced distribution of hydrophobic interactions across its interaction profile. These findings suggest that the various complexes may have different mechanisms of action, depending on the specific interactions they establish with their ligands.

**Table 2:** Protein–ligand interactions of the best pose for each ligand and patterns of interactions between the complexes and their ligands

Complex	Hydrophobic interaction	Hydrogen bond	π^−^stacking	Salt briges
AChE/Anhydrolycorine	5	0	2	0
BuChE/Galanthindole	4	1	1	0
NMDA/Homolycorine	4	3	1	1
GSK3β/Kirkine	6	3	0	0

By identifying the specific interactions between a protein target and their ligands, we can gain valuable insights into the underlying mechanisms of binding and the potential functional effects of these interactions. This knowledge serves as a foundation for designing and optimizing ligands or drugs with enhanced binding properties, ultimately leading to therapeutic outcomes. The molecular interactions identified in the analyzed complexes (Table 2 and Fig. 3), including hydrophobic interactions, hydrogen bonds, π-stacking, and salt bridges, each play distinct roles in stabilizing ligand binding and enhancing specificity. Hydrophobic interactions are predominant across all complexes, acting as the primary mechanism for anchoring ligands within the hydrophobic pockets of the target proteins. Hydrogen bonds, which are critical contributors to binding specificity and stability, were particularly prominent in the NMDA/Homolycorine and GSK3β/Kirkine complexes, highlighting their essential role in molecular recognition. π-Stacking interactions, observed in complexes featuring aromatic residues in the binding site, such as AChE and BuChE, further enhanced stabilization through aromatic-aromatic interactions. Salt bridges identified in the NMDA/Homolycorine complex provided electrostatic complementarity, thereby strengthening the ligand–protein interaction. This understanding of interaction profiles provides a solid foundation for designing ligands tailored to exploit these molecular features. For instance, insights into the predominant hydrophobic interactions or hydrogen bonds within a specific complex facilitate the rational design of ligands optimized for these interactions, potentially leading to more effective binding and superior therapeutic outcomes. Consequently, the results presented in Table 2 and Fig. 3 serve as valuable guidance for the development of novel ligands or drugs that can selectively and effectively target each protein complex. For instance, knowledge of predominant hydrophobic interactions or hydrogen bonds in each complex enables the rational design of ligands optimized for these interactions, leading to more effective binding and potentially superior therapeutic outcomes. Therefore, the results presented in Table 2 and Fig. 3 are valuable for guiding the development of novel ligands or drugs that can selectively and effectively target each protein complex.

To evaluate the potential toxicity of the compounds under investigation, we used the ProTox-II server, and the results are presented in Table 3. This server predicts the likelihood of a compound causing carcinogenic, immunotoxic, mutagenic, and cytotoxic effects, while also estimating the LD50 value (in mg/kg body mass). The LD50 values generated by the server classify molecules based on their toxicity, ranging from Category I (indicating compounds that have the potential to be lethal) to Category VI (indicating compounds with a low risk of harm if ingested) [35]. Although kirkine showed the lowest LD50 value, indicating potential toxicity upon ingestion, it was classified as Category 3, suggesting moderate toxicity. In contrast, the other ligands were categorized as Category 4, indicating a level of toxicity that can be harmful if ingested.

**Table 3:** Prediction of toxicological properties analyzed by ProTox-II

Ligand	Hepatoxicity	Carcinogenicity	Immunotoxicity	Mutagenicity	Cytotoxicity	DL50/probability	Toxicity class
Anhydrolycorine	Inactive (0.88)	Active (0.54)	**Active (0.91)**	Active (0.51)	Inactive (0.57)	1000/0.67	4
Kirkine	Inactive (0.88)	Active (0.50)	Active (0.53)	Inactive (0.71)	Active (0.51)	230/0.68	3
Homolycorine	Inactive (0.90)	Active (0.60)	**Active (0.84)**	Inactive (0.88)	Inactive (0.77)	561/0.68	4
Galanthindole	Inactive (0.69)	Active (0.52)	**Active (0.93)**	Active (0.53)	Inactive (0.62)	525/0.54	4

**Fig. 4:**
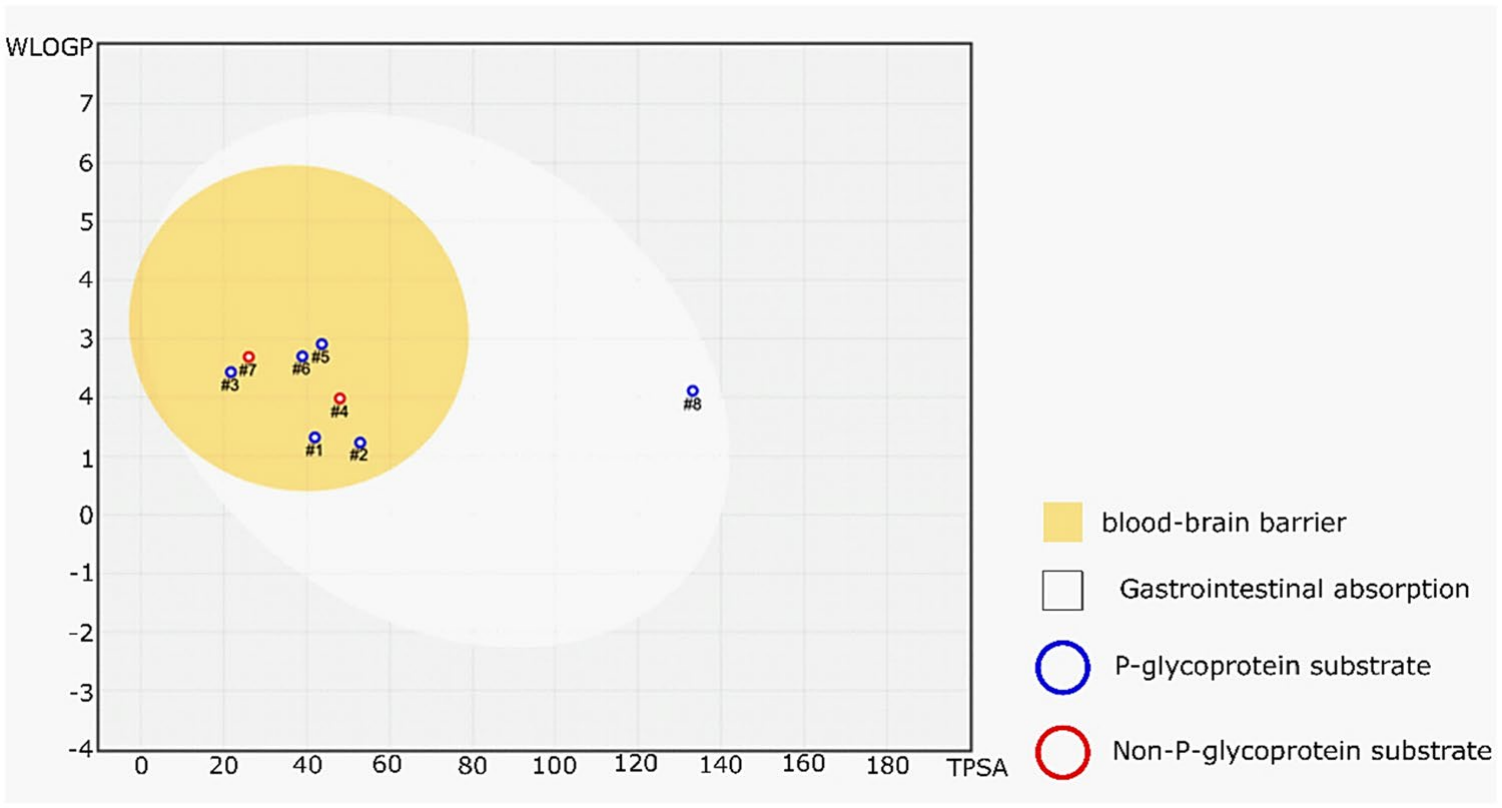
Boiled-egg graphic shows evaluation of passive gastrointestinal absorption (white area) and brain access (yellow area) of the alkaloids and positive-controls: #1Galantamine; #2 Kirkine; #3 anhydrolycorine; #4 homolycorine; #5 galanthindole; #6 tacrine; #7 memantine; #8 6LQ and #9 ATP

Drug development presents a significant challenge for the pharmaceutical industry. It has been demonstrated that the primary factor contributing to the high failure rate of new chemical entities during drug development is not necessarily a lack of drug activity, but rather inadequate pharmacokinetic (PK) and pharmacodynamics (PD) properties. Gastrointestinal absorption and brain access are two critical pharmacokinetic processes that must be evaluated at various stages of drug discovery [59, 60]. Therefore, in addition to assessing the affinity and selectivity against molecular targets, we also evaluated absorption, distribution, metabolism, excretion and tolerable toxicity (ADMET). In the development of anti-Alzheimer drugs, a major limitation is overcoming blood brain barrier (BBB) permeation. The lack of BBB permeability prevents the active compound from reaching its target in the brain. The Brain Or Intestinal Estimated Permeation (BOILED-Egg) method is an accurate predictive model that computes the lipophilicity and polarity of small molecules [59]. The boiled-egg graphic in Fig. 4 presents the results of the assessment of human intestinal absorption (HIA) and BBB penetration. The yellow region indicates a high probability of permeation through the BBB, where seven molecules are located. Homolycorine and memantine are represented as red dots, indicating that they are P-gp non-substrates (P-gp-), whereas galantamine, kirkine, anhydrolycorine, galanthindole, tacrine, and ligand 6LQ are classified as P-gp substrates (P-gp +) (blue dot). It should be noted that ligand 6LQ is in the white region, indicating that it is passively absorbed by the gastrointestinal tract but still a P-gp+. One molecule, ATP, falls outside the range of the graphic, suggesting limited potential for gastrointestinal absorption and BBB penetration. The Log S value of -4.07 indicates that ATP has high water solubility and may be largely confined to extracellular space. The Log P o/w value of 0.35 suggests that ATP has low lipophilicity, which is consistent with the limited potential for HIA and BBB penetration. Considering this, it seems reasonable to conclude that the position of ATP out of range in the BOILED-Egg graphic is consistent with its limited potential for systemic exposure and its properties as a P-gp+ (Table 4).

**Table 4:** Pharmacokinetic properties of the alkaloids tested by the SwissADME Server

	Ligands	Lipinski’s Rule				Solubility (EPSOL)	Lipophilicity	Benchmark
		MW	MLOGP	N or O	NH or OH	Mg/L	Log Po/w	
Clinically Approved Drugs	Galantamine	287.35	1.74	4	1	− 2.93	2.64	FDA—approved [61]
	Tacrine	198.26	2.33	1	1	− 3.27	2.09	FDA—approved [62]
	Memantine	179.30	3.02	1	1	− 3.02	2.51	FDA—approved [63]
Natural Compunds	Kirkine	273.33	1.5	4	2	− 2.25	2.42	None
	Anhydrolycorine	251.28	2.86	2	0	− 3.81	2.63	None
	Homolycorine	315.36	2.15	5	0	− 3.09	2.98	None
	Galanthandiole	281.31	2.09	3	1	− 3.59	2.77	None
GSK3 Reference	6LQ	535.66	0.68	8	2	− 4.07	3.29	Co-crystallized
	ATP	507.18	-4.79	16	7	0.93	0.35	Substrate

^*^The compounds Galantamine, Tacrine, and Memantine were included as references in this study due to their status as FDA-approved drugs, making them reliable benchmarks within a clinical context. Additionally, the compounds 6LQ and ATP were employed as references for the enzyme GSK3. 6LQ was selected because it is co-crystallized with the enzyme, while ATP, as the natural substrate of GSK3, is particularly relevant for comparative analyses of activity and affinity

Lipinski's rule of five, as employed in SwissADME, elucidates the correlation between pharmacokinetic and physicochemical parameters of orally active compounds. This rule characterizes small molecule profiles based on four descriptors: molecular weight (MW) ≤ 500 Da, partition coefficient octanol–water (MLOGP) ≤ 4.15, number of hydrogen bond acceptors (N or O) ≤ 10, and number of hydrogen bond donors (NH or OH) ≤ 5 [59]. (Table 4).

Galantamine adheres to Lipinski's rule of five, exhibiting moderate solubility (2.64 mg/L) and moderate lipophilicity (-2.93 Log P _O/W_). Similarly, Kirkine also conforms to Lipinski's rule, showing moderate solubility (2.42 mg/L) and moderate lipophilicity (-2.25 Log P _O/W_). Anhydrolycorine also complies with the rule demonstrating moderate solubility (2.63 mg/L) and high lipophilicity (-3.81 Log P _O/W_). Homolycorine adheres to Lipinski's rule, with moderate solubility (2.98 mg/L) and moderate lipophilicity (-3.09 Log P _O/W_). Galanthindole, tacrine, and memantine also conform to Lipinski's rule, with moderate solubility values (2.77 mg/L, 2.09 mg/L, and 2.51 mg/L, respectively) and moderate lipophilicity (-3.59 Log P _O/W_, -3.27 Log P _O/W_, and -3.02 Log P _O/W_, respectively). In contrast, 6LQ and ATP do not fully comply with Lipinski's rule. 6LQ exhibits low solubility (3.29 mg/L) and very high lipophilicity (-4.07 Log P _O/W_), while ATP demonstrates high solubility (0.93 mg/L) but low lipophilicity (0.35 Log P _O/W_). Consequently, according to Lipinski's rule, galantamine, kirkine, anhydrolycorine, hololycorine, galanthindole, tacrine, and memantine are considered toconform, while 6LQ and ATP do not. Based on these results, most of the ligands in Table 4 (galantamine, kirkine, anhydrolycorine, hololycorine, galanthindole, tacrine, and memantine) comply with Lipinski's rule. This suggests a higher probability of oral bioavailability for these ligands. The ligands generally exhibit moderate solubility in water, ranging from 2.09 mg/L to 3.29 mg/L. However, it is important to note that 6LQ demonstrates relatively low solubility compared to the other ligands. The lipophilicity of the ligands varies, with 6LQ and galanthindole demonstrating high lipophilicity, while ATP exhibits low lipophilicity. Lipophilicity influences a drug's ability to permeate cell membranes and distribute throughout the body. The molecular weights of the ligands range from 179.30 g/mol to 535.66 g/mol. Larger molecules, such as 6LQ, may encounter challenges in absorption and distribution. In conclusion, ligands that adhere to Lipinski's rule possess moderate solubility, and moderate lipophilicity are more promising candidates for further drug development, with a higher probability of favorable pharmacokinetics properties.

### Molecular Dynamics

**Fig. 5:**
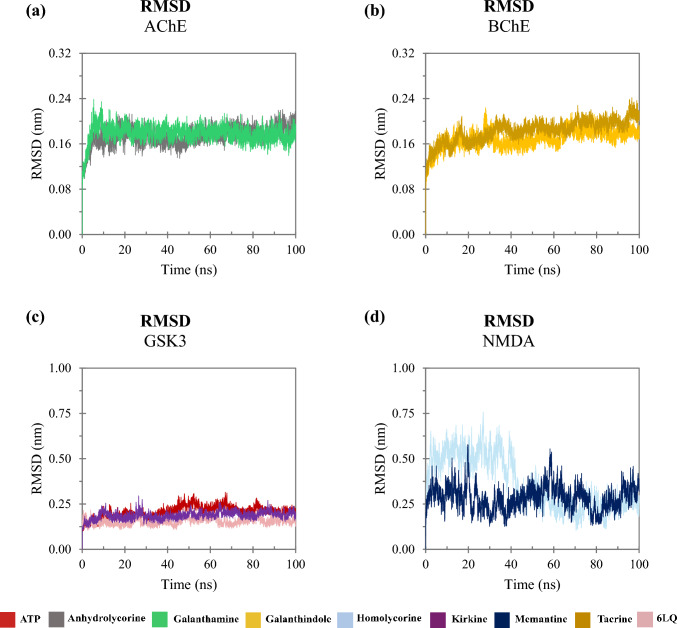
Backbone RMSD as a function time: **a** trajectories of compounds interacting with AChE; **b** with BuChE; **c** with GSK3 and **d** with NMDA

Dynamics simulations were conducted to assess the molecular behavior of the complexes over time. To evaluate the stability of each system, we analyzed the RMSD, which measures the average distance between the atoms of each protein over the simulation frames. Lower RMSD values indicate that the structure remains close to its initial conformation, while higher values suggest significant deviations. A stable system typically shows a plateau in RMSD values, indicating that the complex has reached equilibrium, and its structure is no longer undergoing large fluctuations. Figure 5 illustrates the RMSD of cholinesterases (AChE and BuChE), NMDA and GSK3. The trajectories of these enzymes interacting with the ligands stabilize over the course of the simulation. The complexes involving AChE, BuChE and GSK3 reached stability around 10 ns, while the NMDA system took a longer time to stabilize, with equilibrium achieved around 50 ns. The complexes with cholinesterases and GSK3 exhibited RMSD values ​​close to 0.18 nm (Figs. 5a–c). In contrast, for the NMDA complexes, the homolycorine ligand initially showed high RMSD values, but these values stabilized near 0.25 nm after 50 ns, resembling the behavior of the NMDA-Memantine complex (Fig. 5d).

**Fig. 6:**
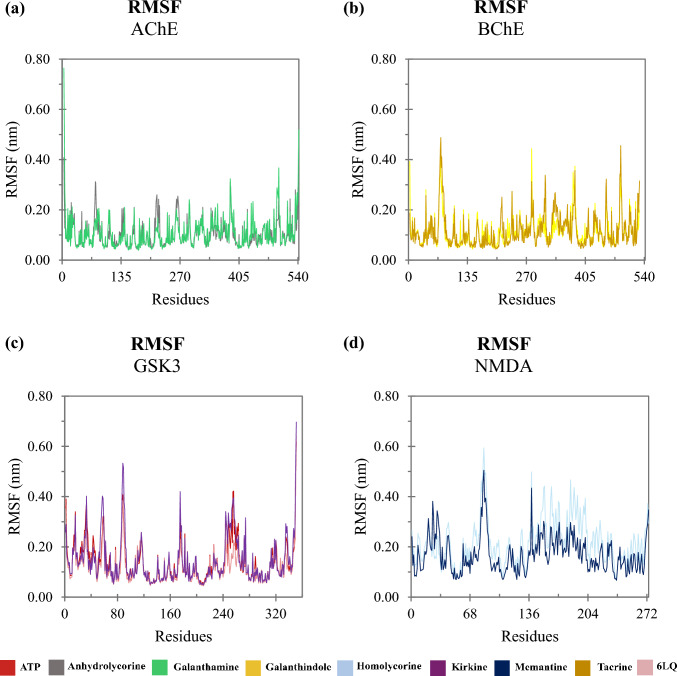
RMSF as function amino acid residues: **a** trajectories of compounds interacting with AChE; **b** with BuChE; **c** with GSK3 and **d** with NMDA

To assess the amino acid residues contributing to fluctuations in trajectories, we analyzed the RMSF of each complex (Fig. 6). RMSF quantifies the flexibility of residues by measuring their average deviation from a reference position over time, highlighting regions of the protein that exhibit greater or lesser mobility. High RMSF values indicate more flexible regions, while low values correspond to more rigid and stable regions. The AChE complexes exhibited similar fluctuation patterns, with notable peaks for residues TYR77 and LEU221 being higher when AChE was associated with the anhydrolycorine ligand (Fig. 6a). For BuChE the complexes with both ligands showed comparable fluctuations throughout the simulation (Fig. 6b). Regarding the GSK3 complexes, the 6LQ ligand induced smaller fluctuations, particularly in the MET250-ILE262 loop (Fig. 6c). In contrast, the NMDA complexes demonstrated higher RMSF values across most amino acid residues with the NMDA-homolycorine complex exhibiting the greatest fluctuation (Fig. 6d).

**Fig. 7:**
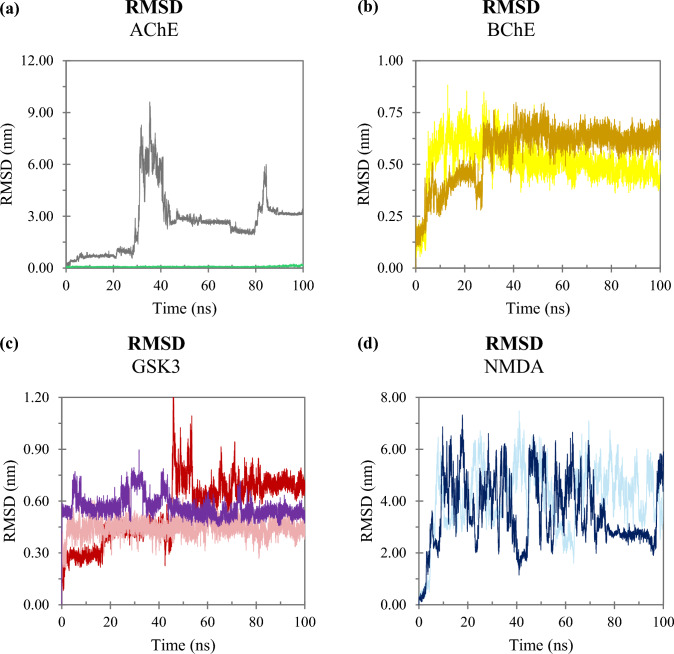
Ligand RMSD as a function time: **a** trajectories of compounds interacting with AChE; **b** witch BuChE; **c** with GSK3; and **d** with NMDA

When analyzing the RMSD of the ligands, we observed that most ligands exhibited RMSD values ​​greater than 0.2 nm across the analyzed enzymes (Fig. 7). The only exception was galantamine interacting with AChE, which displayed a trajectory with peaks below this threshold, stabilizing at approximately 0.08 nm (Fig. 7a). However, the higher RMSD values observed for the other ligands suggest that their positions varied throughout the simulation. In the case of the BuChE complexes, although the trajectories shown peaks above the threshold, the tacrine and galanthindole ligands tend to stabilize at approximately 0.5 and 0.6 nm, respectively (Fig. 7b). Likewise, kirkine, ATP, and 6LQ tend to stabilize around 0.5, 0.7 and 0.4 nm, respectively (Fig. 7c). On the other hand, the trajectories of the AChE-anhydrolycorine complex (Fig. 7a) and of the NMDA complexes (Fig. 7d) exhibited significant fluctuation throughout the simulation, suggesting that these ligands may be subject to dissociation events.

**Fig. 8:**
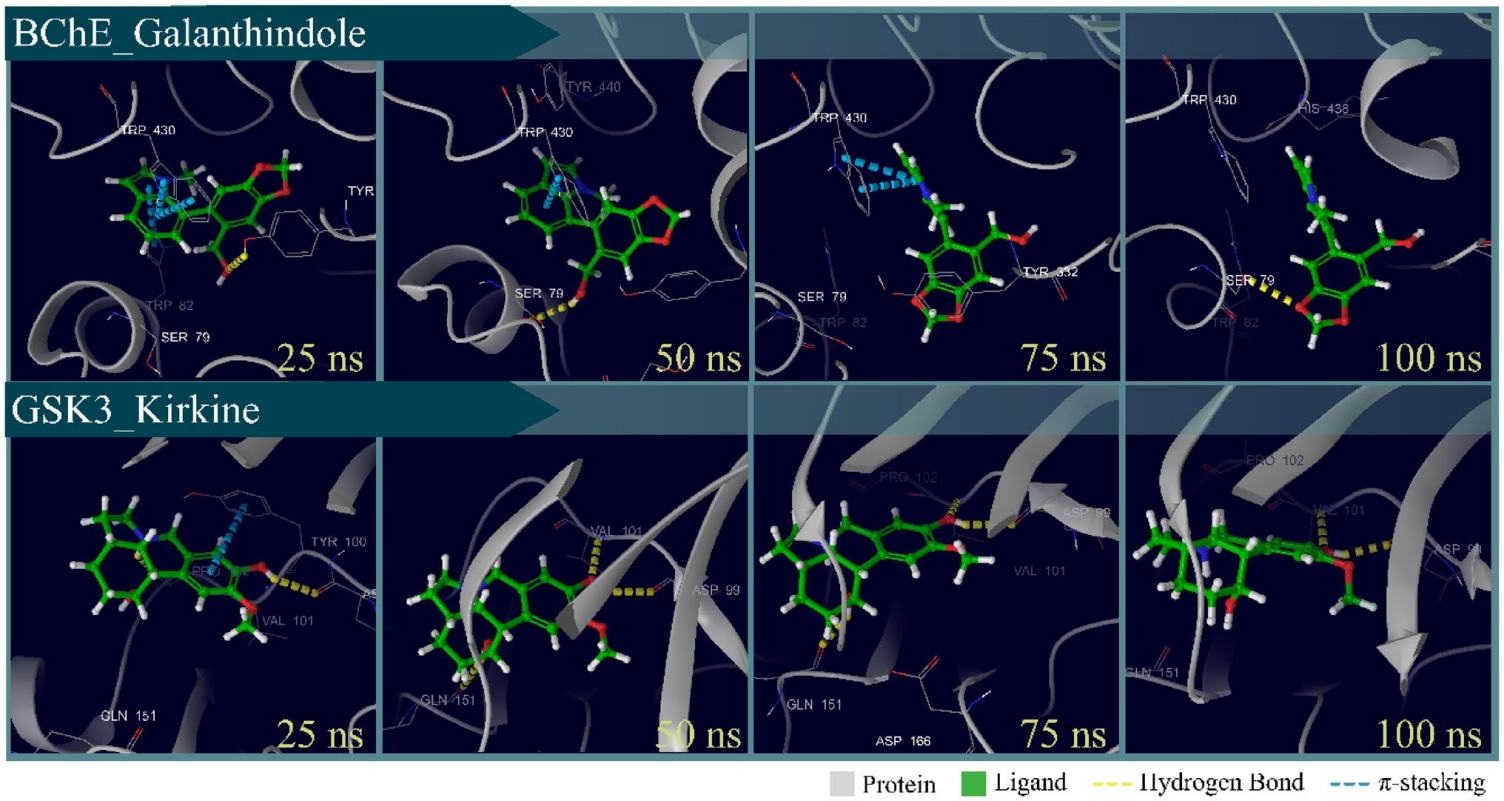
Trajectories of frames of Galanthindole bound with BChE and Kirkine linked with GSK3. Proteins are represented by the color gray in the cartoon format and the ligands have carbon atoms customized in green in the sticks format

Trajectory frames indicated that although the AChE-anhydrolycorine complex and the systems formed by BuChE and GSK3 exhibited RMSD peaks above the 0.2 nm threshold, these ligands remained tightly bound to their respective binding sites. Specifically, galantindole and kirkine, natural products associated with BuChE and GSK3 respectively, showed only minor conformational variations over time. However, they remained anchored within the active sites of these enzymes, stabilized by hydrogen bonding and π-stacking interactions, and surrounded by hydrophobic residues. Figure 8 illustrates representative frames of these ligands. Figure 8 illustrates representative frames of these ligands.

**Fig. 9:**
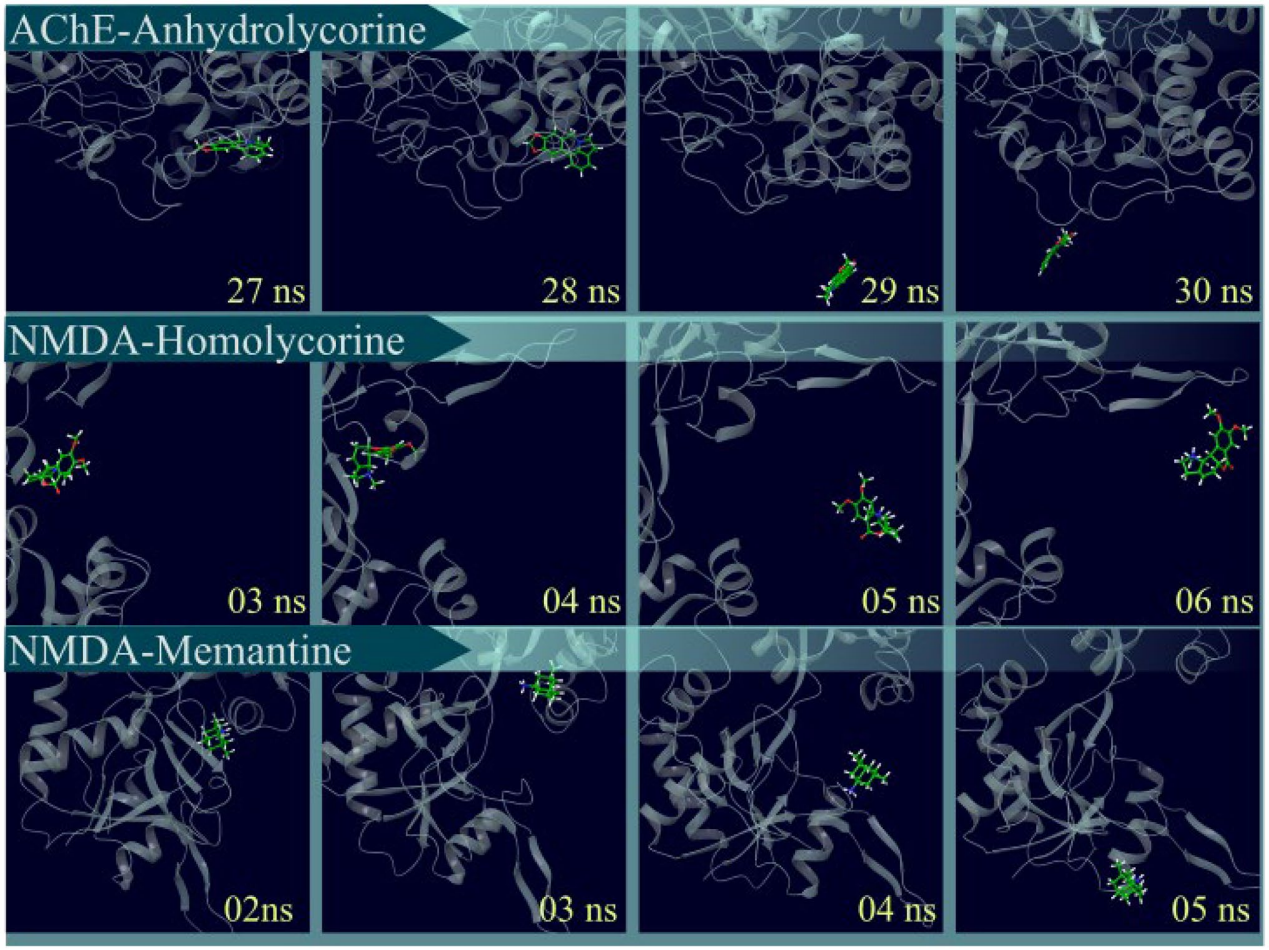
Trajectories of frames of anhydrolycorine bound with AChE and homolycorine and memantine linked with NMDA. Proteins are represented by the color gray in the cartoon format and the ligands have carbon atoms customized in green in the sticks format

In contrast, the trajectories of anhydrolycorine associated with AChE, along with the ligands interacting with the NMDA receptor, showed susceptibility to dissociation events. The dissociation of these ligands occurs at specific time for each complex (Fig. 9. Specifically, anhydrolycorine began to dissociation from the AChE active site at approximately 29 ns, while homolycorine and memantine initiate dissociation from the NMDA site at around 5 ns and 4 ns, respectively. Given that homolycorine and memantine dissociatefrom NMDA and anhydrolycorine leavs AChE site, we proceed to analyze the radius of gyration (Rg), solvent-accessible surface area (SASA), and free energy calculations (MM/PBSA) of those ligands that remained docked to their enzyme.

**Figure10:**
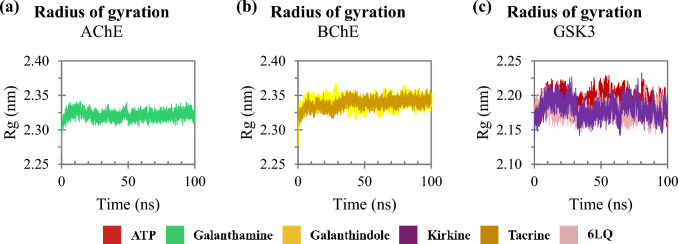
Rg as a function time: **a** trajectories of compounds interacting with AChE; **b** with BuChE and **c** with GSK3

To assess the overall compaction of the proteins that remained interacting with their ligands, the Rg was calculated (Fig. 10). Rg provides information about protein compaction, indicating whether the protein reached conformational equilibrium during the simulations. Figure 10a shows that the trajectory of the AChE-galantamine system equilibrates at approximately 2.32 nm, while Fig. 10b illustrate that the BuChE complexes interacting with galanthindole and tacrine reach Rg values around to 2.34 nm, remaining stable throughout the simulations. This suggests that these protein structures do not undergo significant fluctuation in compaction. Although the GSK3-β complexes exhibited subtle Rg variations (Fig. 10c), the trajectory showed a consistent compaction pattern for the tested ligands. The interaction with kirkine, ATP and the 6LQ presents tenuous compression and decompression movements; however, the trajectories tend to reach equilibrium close to 2.18 nm.

**Fig. 11:**
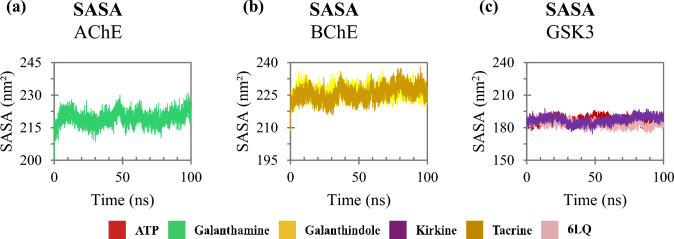
Protein solvent-accessible surface area as a function time: **a** trajectories of compounds interacting with AChE; **b** with BuChE and **c** with GSK3

SASA analysis allows describing the molecular surface of proteins that are accessible to the solvent. The systematic increase in SASA suggests destabilization of the protein, which may expose its hydrophobic regions to the solvent. In general, the SASA values of the AChE, BuChE and GSK3 enzymes stabilized throughout the simulation (Fig. 11). The trajectories of the AChE/anhydrochorine complex stabilized at approximately 219 nm^2^ (Fig. 11a), while the complexes involving the BuChE enzyme demonstrated SASA values close to 226 nm^2^ (Fig. 11b). The GSK3 trajectories demonstrated subtle differences from SASA, where lower values were found when the enzyme interacts with the 6LQ (~ 183 nm^2^) and higher values when it interacts with ATP and kirkine, both with approximately 187 nm^2^ (Fig. 11c).

Through the MM/PBSA analysis, we predicted the binding energies for the ligands that continued to interact with their targets respectively (Table 5). Galantamine was the only compound that remained bound to AChE, showing a binding energy of − 346.30 ± 20.02 kJ.mol^−1^. This strong interaction highlights the potential of galantamine as a lead compound in AChE inhibition, suggesting that its binding profile could serve as a reference for the design of analogues with greater efficacy and selectivity. For the BuChE complexes, the galanthindole ligand exhibited a greater potential to compete for the enzyme´s active site, as it demonstrated a lower binding energy (− 78.15 ± 10.52 kJ.mol^−1^) compared to tacrine (− 68.56 ± 8.28 kJ.mol^−1^). These results highlight the importance of electrostatic interactions in stabilizing galanthindole within the BuChE active site, providing a promising framework for optimizing inhibitors targeting BuChE-related diseases such as AD. Structural modifications that improve these interactions could further enhance binding affinity and specificity. Regarding the GSK3 complexes, kirkine displayed higher binding energy values (− 227.90 ± 10.04 kcal.mol^−1^) compared to ATP and the 6LQ, which had scores of − 881.96 ± 26.27 and − 351.19 ± 13.12 kJ.mol^−1^, respectively. Although Kirkine binding energy is less favorable than ATP, its distinct interaction profile may inspire the design of GSK3 inhibitors capable of selectively modulating its activity, potentially minimizing off-target effects. These findings suggest that MM/PBSA-derived insights into binding energy, together with structural characterization of key interactions, can guide rational drug design by identifying functional groups or moieties essential for binding.

**Table 5:** Residual decomposition and Binding energy (given in kJ mol^−1^) in the protein–ligand interaction

Receptor	Ligand	ΔEvdW	ΔEelec	Polar solvatation energy	SASA energy	Binding energy
AChE	Galantamine	− 149.76 ± 12.01	− 497.33 ± 26.33	317.46 ± 35.55	− 16.66 ± 0.75	− 346.30 ± 20.02
BuChE	Galanthindole	− 115.50 ± 9.45	− 22.22 ± 8.10	72.59 ± 14.87	− 13.01 ± 1.11	− 78.15 ± 10.52
	Tacrine	− 106.61 ± 5.80	− 6.93 ± 3.71	56.51 ± 10.11	− 11.54 ± 0.85	− 68.56 ± 8.28
GSK3	Kirkine	− 124.40 ± 9.02	− 188.15 ± 9.62	99.73 ± 2.99	− 15.09 ± 0.49	− 227,90 ± 10.04
	ATP	− 74.58 ± 14.14	− 1986.59 ± 33.25	1193.01 ± 23.15	− 13.80 ± 0.38	− 881.96 ± 26.27
	6LQ	− 193.40 ± 7.85	− 348.62 ± 11.43	209.59 ± 14.86	− 18.76 ± 0.54	− 351.19 ± 13.12

Taken together, these in vitro and in silico results provide evidence for the neuroprotective effects exerted by plants. Among the Amaryllidaceae family, galantamine-type alkaloids are widely recognized for their potential utility in treatment AD. In recent years, the multifactorial nature of AD has driven an active search for multi-target drugs with two or more selected biological activities, as they could represent a significant pharmacological advancement in managing this complex disease. In this context, drug combinations or compounds that may act at different levels of the neurotoxic cascade may offer new hope for treating neurodegenerative diseases like AD [64–66]. The findings from our study regarding the neuroprotective effects of *C. subedentata* extract align with the current trends in drug discovery for AD, which increasingly emphasize a multi-target approach over the traditional single-target strategies. This approach recognizes the multifactorial nature of AD, where a combination of neurodegenerative processes, such as cholinergic deficits, amyloid beta accumulation, and neuroinflammation, must be addressed simultaneously for more effective treatment outcomes. However, one of the substantial challenges of developing multi-target therapies is ensuring adequate permeability across the BBB [67, 68]. Many promising compounds, including various alkaloids, may exhibit limited bioavailability due to their molecular size and hydrophilicity. Therefore, future research must not only focus on enhancing the efficacy of these multi-target interactions but also on improving the pharmacokinetic properties of the compounds to facilitate their transport across the BBB, ultimately leading to tangible therapeutic benefits in patients suffering from Alzheimer's disease [69, 70]. The findings of this study demonstrate that certain alkaloids significantly inhibit enzyme activity, correlating with docking simulations that reveal strong binding affinities and stable molecular interactions. Combining experimental data and computational predictions enhances understanding of these compounds' neuroprotective effects, paving the way for the rational design of new therapeutic agents targeting Alzheimer's pathology. However, to elucidate the therapeutic potential of *C. subedentata* extract, it is important to investigate the possible synergistic effects between the identified alkaloids and other non-alkaloid metabolites present in the extract. Preliminary studies indicate that the complex phytochemical profile of *C. subedentata* may enhance neuroprotective properties through multi-target interactions. For instance, flavonoids and phenolic compounds, recognized for their antioxidant and anti-inflammatory effects, could synergistically interact with alkaloids to regulate oxidative stress and neuroinflammation associated with Alzheimer's disease. Future research should prioritize the isolation of these non-alkaloid components and the evaluation of their interactions both in vitro and in vivo, as this may lead to a more comprehensive understanding of the extract's overall efficacy and facilitate the development of combination therapies that take advantage of these synergistic interactions.

## Conclusions

Our results demonstrated that *C. subedentata* extract exhibited neuroprotective effects against neurotoxic stimuli induced by Aβ (1–42) peptide and Okadaic acid. Pre-treatments with the extract significantly increased cell survival rates against these neurotoxic challenges, suggesting its potential role in mitigating neuronal death associated with Alzheimer's disease. In terms of in silico approaches, our findings for the first time describe the possible mechanisms of interaction exerted by some alkaloids from *C. subedentata* with key target proteins involved in Alzheimer’s disease- However, it is important to consider the possibility that the neuroprotective mechanisms of this plant may also involve other non-alkaloid compounds. Further research is needed to explore the roles of non-alkaloid metabolites of *C. subedenta* in its neuroprotective effects.

## Future Research Directions

Building upon the promising neuroprotective effects demonstrated by *C. subedentata* extract, several avenues for future research can be pursued to further elucidate its therapeutic potential:

1. Exploration of Non-Alkaloid Metabolites: While our study highlights the role of alkaloids in conferring neuroprotection, it is crucial to investigate other non-alkaloid metabolites present in *C. subedentata*. Future studies should focus on isolating and characterizing these compounds to determine their individual contributions to neuroprotection and their mechanisms of action.
2. In vivo studies: Validate the in vitro findings through animal models of neurodegeneration, such as Alzheimer's disease, to assess the extract's efficacy in reducing cognitive decline and neuronal loss. Additionally, studies could investigate the pharmacokinetics of its active compounds and elucidate the specific molecular mechanisms behind its neuroprotective effects, with a particular focus on neuroinflammation, oxidative stress, apoptosis, and interactions with relevant target proteins.
3. Combination Therapies: Explore the potential synergistic effects of combining *C. subedentata* extract with other therapeutic agents to enhance neuroprotection and treatment outcomes.
4. Clinical Trials: Conducting clinical trials to evaluate the safety, tolerability, and efficacy of *C. subedentata* extract in patients with neurodegenerative diseases, which could pave the way for its potential use as a therapeutic agent. By pursuing these research directions, we can deepen our understanding of *C. subedentata* extract and its constituents, potentially leading to novel strategies for the prevention and treatment of neurodegenerative diseases.

## Data Availability

No datasets were generated or analysed during the current study.

## References

[1] Prince MJ (2015) World Alzheimer Report 2015: the global impact of dementia: an analysis of prevalence, incidence, cost and trends. Alzheimer's Disease International

[2] Sayas CL, Ávila J (2021) GSK-3 and Tau: a key duet in Alzheimer’s disease. Cells 10(4):721 33804962 10.3390/cells10040721 PMC8063930

[3] Anand R, Gill KD, Mahdi AA (2014) Therapeutics of Alzheimer’s disease: past, present and future. Neuropharmacology 76:27–50 23891641 10.1016/j.neuropharm.2013.07.004

[4] Rosenblum WI (2014) Why Alzheimer trials fail: removing soluble oligomeric beta amyloid is essential, inconsistent, and difficult. Neurobiol Aging 35(5):969–974 24210593 10.1016/j.neurobiolaging.2013.10.085

[5] Selkoe, D.J., Preventing Alzheimer’s disease. Science, 2012. 337(6101). 10.1126/science.1228541 22997326

[6] Mintzer JE, Mirski DF, Hoernig KS (2000) Behavioral and psychological signs and symptoms of dementia: a practicing psychiatrist’s viewpoint. Dialogues Clin Neurosci 2(2):139–155 22034243 10.31887/DCNS.2000.2.2/jmintzer PMC3181597

[7] Kumar A, Singh A (2015) A review on Alzheimer’s disease pathophysiology and its management: an update. Pharmacol Rep 67(2):195–203 25712639 10.1016/j.pharep.2014.09.004

[8] Hampel H et al (2017) Revisiting the cholinergic hypothesis in Alzheimer’s disease: emerging evidence from translational and clinical research. J Prev Alzheimer’s Dis 6:2–1510.14283/jpad.2018.4330569080

[9] Anand P, Singh B (2013) A review on cholinesterase inhibitors for Alzheimer’s disease. Arch Pharmacal Res 36(4):375–399 10.1007/s12272-013-0036-3 23435942

[10] Purgatorio R et al (2019) Investigating 1, 2, 3, 4, 5, 6-hexahydroazepino [4, 3-b] indole as scaffold of butyrylcholinesterase-selective inhibitors with additional neuroprotective activities for Alzheimer’s disease. Eur J Med Chem 177:414–424 31158754 10.1016/j.ejmech.2019.05.062

[11] Darvesh S (2016) Butyrylcholinesterase as a diagnostic and therapeutic target for Alzheimer’s disease. Curr Alzheimer Res 13(10):1173–1177 27040140 10.2174/1567205013666160404120542

[12] Riedel G, Platt B, Micheau J (2003) Glutamate receptor function in learning and memory. Behav Brain Res 140(1–2):1–47 12644276 10.1016/s0166-4328(02)00272-3

[13] Hardingham GE, Bading H (2010) Synaptic versus extrasynaptic NMDA receptor signalling: implications for neurodegenerative disorders. Nat Rev Neurosci 11(10):682–696 20842175 10.1038/nrn2911 PMC2948541

[14] Parsons MP, Raymond LA (2014) Extrasynaptic NMDA receptor involvement in central nervous system disorders. Neuron 82(2):279–293 24742457 10.1016/j.neuron.2014.03.030

[15] Ni R, Marutle A, Nordberg A (2013) Modulation of α7 nicotinic acetylcholine receptor and fibrillar amyloid-β interactions in Alzheimer’s disease brain. J Alzheimer’s Dis 33(3):841–851 23042213 10.3233/JAD-2012-121447

[16] Müller-Schiffmann A, Sticht H, Korth C (2012) Hybrid compounds. BioDrugs 26(1):21–31 22239618 10.2165/11597630-000000000-00000

[17] Zheng H, Fridkin M, Youdim M (2015) New approaches to treating Alzheimer’s disease. Perspect Med Chem 7 10.4137/PMC.S13210 PMC4327405 25733799

[18] Elgorashi EE, Stafford GI, Van Staden J (2004) Acetylcholinesterase enzyme inhibitory effects of amaryllidaceae alkaloids. Planta Med 70(3):260–262 15114506 10.1055/s-2004-818919

[19] Eriksson AH et al (2012) In-vitro evaluation of the P-glycoprotein interactions of a series of potentially CNS-active Amaryllidaceae alkaloids. J Pharm Pharmacol 64(11):1667–1677 23058055 10.1111/j.2042-7158.2012.01536.x

[20] Bastida Armengol J et al (2011) In: Muñoz-Torrero D (eds) Chemical and biological aspects of Amaryllidaceae alkaloids, Chapter 3. Recent advances in pharmaceutical sciences, pp 65–100

[21] Heinrich M, Lee Teoh H (2004) Galanthamine from snowdrop—the development of a modern drug against Alzheimer’s disease from local Caucasian knowledge. J Ethnopharmacol 92(2):147–162 15137996 10.1016/j.jep.2004.02.012

[22] Samochocki M et al (2003) Galantamine is an allosterically potentiating ligand of neuronal nicotinic but not of muscarinic acetylcholine receptors. J Pharmacol Exp Ther 305(3):1024–1036 12649296 10.1124/jpet.102.045773

[23] Wang D et al (2007) The allosteric potentiation of nicotinic acetylcholine receptors by galantamine ameliorates the cognitive dysfunction in beta amyloid25–35 icv-injected mice: involvement of dopaminergic systems. Neuropsychopharmacology 32(6):1261–1271 17133263 10.1038/sj.npp.1301256

[24] Cabezas F et al (2013) Análisis del contenido alcaloidico de Caliphruria Subdentata Baker (Amaryllidaceae) por el método CG-EM. Revista latinoamericana de química 41(1):68–73

[25] Castillo WO et al (2018) *Caliphruria subedentata* (Amaryllidaceae) decreases genotoxicity and cell death induced by β-amyloid peptide in sh-sy5y cell line. Mutat Res/Genet Toxicol Environ Mutagen 836:54–61 30442346 10.1016/j.mrgentox.2018.06.010

[26] Gore M, Desai NS (2014) Computer-aided drug designing. Clin Bioinform 10.1007/978-1-4939-0847-9_18 24870144

[27] dos Santos RN, Ferreira LG, Andricopulo AD (2018) Practices in molecular docking and structure-based virtual screening. Comput Drug Discov Des 10.1007/978-1-4939-7756-7_3 29594766

[28] Hoffmann LF et al (2023) Neural regeneration research model to be explored: SH-SY5Y human neuroblastoma cells. Neural Regener Res 18(6):1265–1266 10.4103/1673-5374.358621 PMC9838160 36453406

[29] Bell M, Zempel H (2022) SH-SY5Y-derived neurons: a human neuronal model system for investigating TAU sorting and neuronal subtype-specific TAU vulnerability. Rev Neurosci 33(1):1–15 33866701 10.1515/revneuro-2020-0152

[30] Ates G et al (2017) Assaying cellular viability using the neutral red uptake assay, in Cell Viability Assays 10.1007/978-1-4939-6960-9_2 28470514

[31] Berman H et al (2000) The protein data Bank nucleic acids research 28: 235–242 10.1093/nar/28.1.235 PMC102472 10592235

[32] Jones G et al (1997) Development and validation of a genetic algorithm for flexible docking. J Mol Biol 267(3) 10.1006/jmbi.1996.0897 9126849

[33] Kim S et al (2015) PubChem substance and compound databases. Nucleic Acids Res. 44(D1) 10.1093/nar/gkv951 PMC4702940 26400175

[34] Daina, A., O. Michielin, and V. Zoete, SwissADME: a free web tool to evaluate pharmacokinetics, drug-likeness and medicinal chemistry friendliness of small molecules. Scientific reports, 2017. 7. 10.1038/srep42717 PMC5335600 28256516

[35] Banerjee P et al (2018) ProTox-II: a webserver for the prediction of toxicity of chemicals. Nucleic Acids Res 46(W1) 10.1093/nar/gky318 PMC6031011 29718510

[36] Salentin S et al (2015) PLIP: fully automated protein–ligand interaction profiler. Nucleic Acids Res 43(W1) 10.1093/nar/gkv315 PMC4489249 25873628

[37] Van Der Spoel D et al (2005) GROMACS: fast, flexible, and free. Journal of computational chemistry 26(16) 10.1002/jcc.20291 16211538

[38] Huang J et al (2017) CHARMM36m: an improved force field for folded and intrinsically disordered proteins. Nat Methods 14(1) 10.1038/nmeth.4067 PMC5199616 27819658

[39] Hanwell MD et al (2012) Avogadro: an advanced semantic chemical editor, visualization, and analysis platform. J Cheminform, p 4 10.1186/1758-2946-4-17 PMC3542060 22889332

[40] Vanommeslaeghe K et al (2010) CHARMM general force field: a force field for drug-like molecules compatible with the CHARMM all-atom additive biological force fields. J Comput Chem 31(4):671–690 19575467 10.1002/jcc.21367 PMC2888302

[41] Kumari R et al (2014) g_mmpbsa—a GROMACS tool for high-throughput MM-PBSA calculations. J Chem Inform Model 54(7):1951–1962 10.1021/ci500020m 24850022

[42] Yee A et al (2018) Alzheimer’s disease: insights for risk evaluation and prevention in the Chinese population and the need for a comprehensive programme in Hong Kong/China. Hong Kong Med J 24(5):492–500 30232267 10.12809/hkmj187244

[43] Lee SL, Thomas P, Fenech M (2014) Extracellular amyloid beta 42 causes necrosis, inhibition of nuclear division, and mitotic disruption under both folate deficient and folate replete conditions as measured by the cytokinesis-block micronucleus cytome assay. Environ Mol Mutagen 55(1):1–14 24038346 10.1002/em.21811

[44] Narciso L et al (2016) The response to oxidative DNA damage in neurons: mechanisms and disease. Neural Plast 2016:3619274 26942017 10.1155/2016/3619274 PMC4752990

[45] Castillo WO et al (2016) Galanthamine decreases genotoxicity and cell death induced by β-amyloid peptide in SH-SY5Y cell line. Neurotoxicology 57:291–297 27793617 10.1016/j.neuro.2016.10.013

[46] Musalmah M, Rusdiah RJ, Noor Aini AH (2009) Induction of DNA damage and cell death by beta amyloid peptide and its modification by tocotrienol rich fraction (TRF). Med Health 4(1):8–15

[47] Yoon SY et al (2005) Inactivation of GSK-3β in okadaic acid-induced neurodegeneration: relevance to Alzheimer’s disease. NeuroReport 16(3):223–227 15706224 10.1097/00001756-200502280-00004

[48] Del Barrio L et al (2011) Neurotoxicity induced by okadaic acid in the human neuroblastoma SH-SY5Y line can be differentially prevented by α7 and β2* nicotinic stimulation. Toxicol Sci 123(1):193–205 21715663 10.1093/toxsci/kfr163

[49] Metin-Armağan D et al (2018) Okadaic acid–induced tau hyperphosphorylation and the downregulation of Pin1 expression in primary cortical neurons. J Chem Neuroanat 92:41–47 29860071 10.1016/j.jchemneu.2018.05.006

[50] Wang HB et al (2018) ERα36 gene silencing promotes tau protein phosphorylation, inhibits cell proliferation, and induces apoptosis in human neuroblastoma SH-SY5Y cells. FASEB J 32(12):6456–6468 10.1096/fj.201701386 29932870

[51] Atasoy İL et al (2017) Both secreted and the cellular levels of BDNF attenuated due to tau hyperphosphorylation in primary cultures of cortical neurons. J Chem Neuroanat 80:19–26 27914953 10.1016/j.jchemneu.2016.11.007

[52] Zhao L et al (2016) Influence of okadaic acid on hyperphosphorylation of tau and nicotinic acetylcholine receptors in primary neurons. Exp Biol Med 241(16):1825–1833 10.1177/1535370216650759 PMC5027944 27190248

[53] Zhang H et al (2020) Tolfenamic acid inhibits GSK-3β and PP2A mediated tau hyperphosphorylation in Alzheimer’s disease models. J Physiol Sci 70:1–11 32517647 10.1186/s12576-020-00757-y PMC10717460

[54] Saito T et al (2019) Early administration of galantamine from preplaque phase suppresses oxidative stress and improves cognitive behavior in APPswe/PS1dE9 mouse model of Alzheimer’s disease. Free Radic Biol Med 145:20–32 31536772 10.1016/j.freeradbiomed.2019.09.014

[55] Course MM, Wang X (2016) Transporting mitochondria in neurons. F1000Research 10.12688/f1000research.7864.1 PMC4955021 27508065

[56] Simões RF et al (2021) Refinement of a differentiation protocol using neuroblastoma SH-SY5Y cells for use in neurotoxicology research. Food Chem Toxicol 149:111967 33417974 10.1016/j.fct.2021.111967

[57] Castillo Ordoñez WO et al (2023) Epigenetic regulation exerted by Caliphruria subedentata and galantamine: an in vitro and in silico approach for mimic Alzheimer’s disease. J Biomol Struct Dyn 42:11215–11230 37814967 10.1080/07391102.2023.2261034

[58] Qiu C, Kivipelto M, von Strauss E (2009) Epidemiology of Alzheimer’s disease: occurrence, determinants, and strategies toward intervention. Dialogues Clin Neurosci 11(2):111–128 19585947 10.31887/DCNS.2009.11.2/cqiu PMC3181909

[59] Daina A, Zoete V (2016) A boiled-egg to predict gastrointestinal absorption and brain penetration of small molecules. ChemMedChem 11(11):1117–1121 27218427 10.1002/cmdc.201600182 PMC5089604

[60] Passeleu-Le Bourdonnec C et al (2013) Methodologies to assess drug permeation through the blood–brain barrier for pharmaceutical research. Pharm Res 30(11):2729–2756 23801086 10.1007/s11095-013-1119-z

[61] Thompson C (2001) A, FDA approves galantamine for Alzheimer’s disease. Am J Health-Syst Pharm: AJHP: Off J Am Soc Health-Syst Pharm 58(8):649 10.1093/ajhp/58.8.649a 11329755

[62] Crismon ML (1994) Tacrine: first drug approved for Alzheimer’s disease. Ann Pharmacother 28(6):744–751 7919566 10.1177/106002809402800612

[63] Ferris S (2003) H, Evaluation of memantine for the treatment of Alzheimer’s disease. Expert Opin Pharmacother 4(12):2305–2313 14640929 10.1517/14656566.4.12.2305

[64] Romero A et al (2010) Synergistic neuroprotective effect of combined low concentrations of galantamine and melatonin against oxidative stress in SH-SY5Y neuroblastoma cells. J Pineal Res 49(2):141–148 20536682 10.1111/j.1600-079X.2010.00778.x

[65] Bolognesi ML et al (2009) Alzheimer’s disease: new approaches to drug discovery. Curr Opin Chem Biol 13(3):303–308 19467915 10.1016/j.cbpa.2009.04.619

[66] Agis-Torres A et al (2014) Multi-target-directed ligands and other therapeutic strategies in the search of a real solution for Alzheimer’s disease. Curr Neuropharmacol 12(1):2–36 24533013 10.2174/1570159X113116660047 PMC3915347

[67] Soltan, Osama M et al (2024) Design of multi-target drugs of HDACs and other anti-alzheimer related targets: current strategies and future prospects in Alzheimer’s diseases therapy. Bioorg Chem 107651 10.1016/j.bioorg.2024.107651 39029320

[68] Pathak C, Kabra UDA (2024) Comprehensive review of multi-target directed ligands in the treatment of Alzheimer’s disease. Bioorg Chem 107152 10.1016/j.bioorg.2024.107152 38290187

[69] Niazi SK, Magoola M, Mariam Z (2024) Innovative therapeutic strategies in Alzheimer’s disease: a synergistic approach to neurodegenerative disorders. Pharmaceuticals 17(6):741 38931409 10.3390/ph17060741 PMC11206655

[70] Tang P (2023) Challenges and opportunities for improving the druggability of natural product: Why need drug delivery system? Biomed Pharmacother 164:1–22 10.1016/j.biopha.2023.114955 37269810

